# Extraction of Fish Bones Embedded in the Esophagus *via* Endoscopic Submucosal Dissection: Two Case Reports and Literature Review

**DOI:** 10.3389/fmed.2021.746720

**Published:** 2021-10-29

**Authors:** Dan Lu, Lu Lv, Qing Gu, Ajay Jain, Björn Berglund, Feng Ji

**Affiliations:** ^1^Department of Endoscopy Center, The First Affiliated Hospital, College of Medicine, Zhejiang University, Hangzhou, China; ^2^Department of Gastroenterology, The First Affiliated Hospital, College of Medicine, Zhejiang University, Hangzhou, China; ^3^Meridian Medical Group, Indiana University Health Methodist Hospital, Indianapolis, IN, United States; ^4^Department of Biomedical and Clinical Sciences, Linköping University, Linköping, Sweden; ^5^State Key Laboratory for Diagnosis and Treatment of Infectious Diseases, Collaborative Innovation Center for Diagnosis and Treatment of Infectious Diseases, The First Affiliated Hospital, College of Medicine, Zhejiang University, Hangzhou, China

**Keywords:** foreign body, esophagus, migration, endoscopic submucosal dissection, case report

## Abstract

Foreign body ingestion is a common problem encountered at gastroenterology clinics and emergency rooms which can cause serious complications. Usually, foreign bodies are directly visible with flexible endoscopes and can be readily removed. However, when foreign bodies migrate into the deeper tissue of the esophagus, surgery is typically required. There is currently no consensus regarding the best treatment. In this report, we present two cases in which fish bones embedded in the submucosal and muscularis propria of the esophagus were successfully removed *via* endoscopic submucosal dissection (ESD). Both patients were discharged without any complications.

## Introduction

Foreign bodies in the esophagus are commonly encountered in cases at gastroenterology clinics or in emergency rooms. Patients with foreign bodies impacted in the esophagus can present with dysphagia, and/or odynophagia (1). Most foreign bodies are visualized directly with flexible endoscopes and can be readily removed. However, sharp and needle-like foreign bodies, such as fish bones, increase the risk of migration into the submucosa or into the deeper layer in the gastrointestinal tract. Although very rarely, this can lead to serious complications, including stricture formation, esophageal perforation, tracheoesophageal fistulas and aortoesophageal fistulas. If the diagnosis is considerably delayed, the complications can even be fatal (2). As buried fish bones cannot be endoscopically recognized, localization and removal become challenging with conventional endoscopic methods. Indeed, interventions involving extensive surgical dissection and substantial tissue trauma are often necessary. To date, there is no consensus regarding the best treatment.

Endoscopic submucosal dissection (ESD) was developed primarily for early gastric cancer, or submucosal lesions (3). We successfully applied ESD in order to remove fish bones embedded in the esophagus in two recent cases. ESD is less invasive compared to surgery (4). Herein, we describe our experience managing esophageal foreign body by using the ESD technique and discuss the results. To the best of our knowledge, there has to date been no similar case reports describing using ESD to remove fish bones buried within the muscularis propria layer of the esophagus.

## Case Presentation

### Case 1

**Figure 1:**
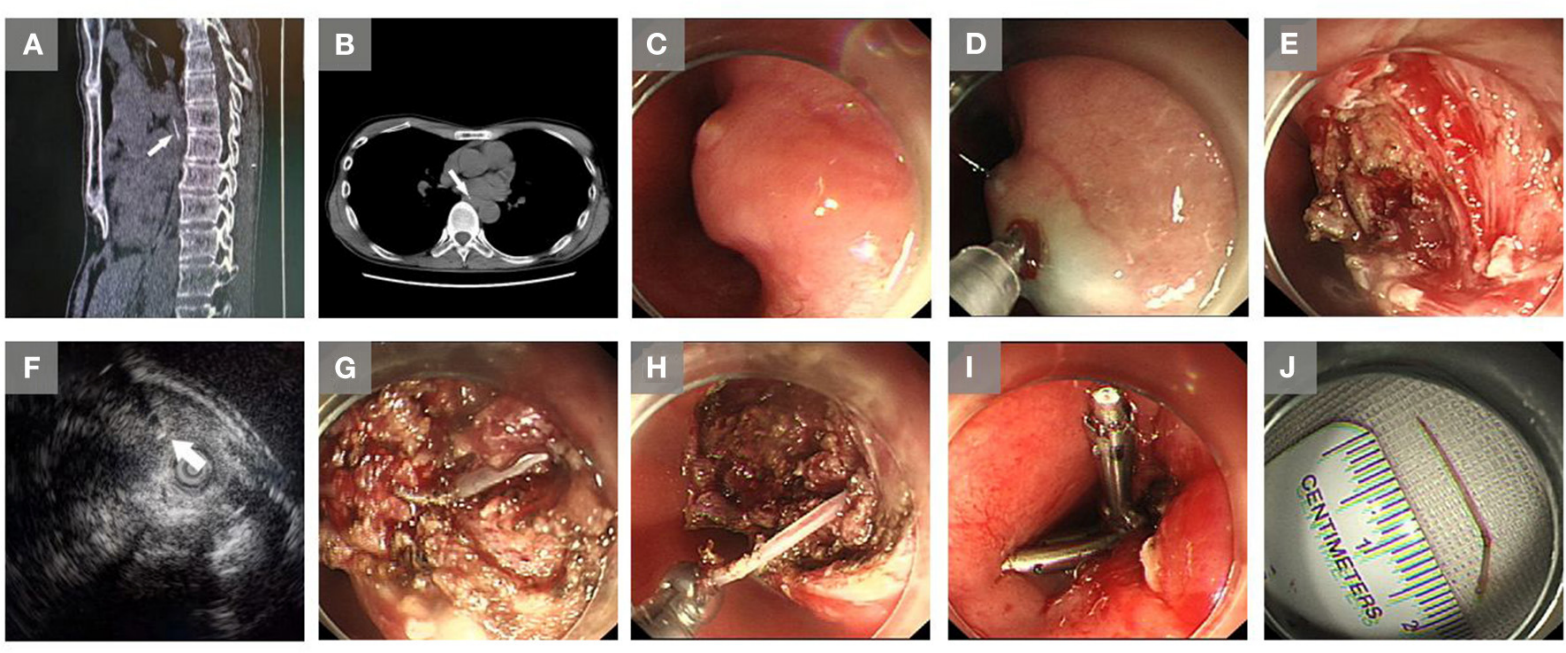
Images of the embedded foreign body in the esophagus, and extraction *via* endoscopic submucosal dissection in Case 1. **(A)** A buried foreign body (arrow) in the esophagus was revealed by a computer tomography (CT) scan at the local hospital. **(B)** A buried foreign body (arrow) in the esophagus was revealed by the CT scan at our hospital. **(C)** A mucosal protrusion with a small ulcer was observed with endoscopy. **(D)** A submucosal injection was performed around the mucosal protrusion. **(E)** Dissection of the mucosa and submucosa. **(F)** Endoscopic ultrasound imaging showed a fish bone-like linear hyperechoic mass. **(G)** Entirely exposed foreign body (a fish bone). **(H)** Removal of the foreign body (a fish bone) by forceps. **(I)** The wound was closed by using two metal clips. **(J)** The removed fish bone.

A sixty-five-year-old man presented at the local hospital with severe retrosternal chest pain after having swallowed a fish bone 1 week earlier. The foreign body was not discovered with conventional gastroscopy at the local hospital. The pain subsided approximately seven days later. After an additional week, the patient once more visited the local hospital after the retrosternal chest pain had reoccurred. An esophageal computed tomography (CT) revealed a foreign body buried in the submucosa of the esophagus (Figure 1A) and an endoscopic diagnosis of esophageal ulcer and bile reflux gastritis was made during this second gastroscopy.

The patient was then transferred to our hospital. His medical and family history was unremarkable. The temperature was 36.8°C. Other vital signs were stable on admission. The white blood cell count was 3.41 × 10^9^/L (reference range 4.0–10.0 × 10^9^/L), with 74.2% neutrophils (reference range 50–70%), and the C-reactive protein level was 0.4 mg/L (reference range 0.0–8.0 mg/L). Other laboratory results were normal. A second esophageal CT showed a linear, high-density foreign body embedded at the level of the eighth thoracic vertebra, in the wall of the esophagus, edge clear (Figure 1B). Considering the foreign body had migrated into the deeper layer of the esophagus, the patient was arranged for subsequent ESD. The procedure was conducted under general anesthesia with endotracheal intubation. A mucosal protrusion with a small ulcer on it was identified in the proximal esophagus (Figure 1C). A fluid mixture, which consisted of glycerol-fructose injection (250 ml) and epinephrine (2 mg), stained with methylene blue, was injected under the mucosal protrusion (Figure 1D). A fluid cushion was made to enhance visualization and decrease the risk of perforation. Subsequently, a mucosal incision was made by using a dual knife (KD-650 L; Olympus, Tokyo, Japan) to facilitate entry into the submucosa (Figure 1E). Endoscopic ultrasound (EUS) was then applied by using an Olympus US EU-ME2 PREMIER PLUS (Olympus), a MAJ-1720 sheathed 12-MHz linear-array matrix transducer (Olympus) and an UM-DP20-25R ultrasonic probe (Olympus), to detect the depth of the foreign body migration. The EUS showed that the foreign body had migrated from the entry point into the muscularis propria layer (Figure 1F). The muscularis propria layer was cut open by using a dual knife (Olympus). The foreign body was entirely exposed from its proximal to distal edge (Figure 1G), and was removed with Rat Tooth forceps (Olympus) (Figure 1H). Finally, since the muscularis propria layer was cut, clips were deployed to minimize the risk of post-procedural perforation (Figure 1I). The removed fish bone was 2 cm in length (Figure 1J). On post-procedure day 1, the temperature was 37.3°C, and other vital signs were stable. The white blood cell count was 3.91 × 10^9^/L (reference range 4.0–10.0 × 10^9^/L), with 79.9% neutrophils (reference range 50–70%), and the C-reactive protein level was 18.6 mg/L (reference range 0.0–8.0 mg/L). The patient complained of mild retrosternal pain (one out of 10 on the pain scale) postoperatively. The patient received antiemetic (metoclopramide) for the prevention of postoperative nausea and vomiting, and proton pump inhibitor (omeprazole) for the suppression of gastric acid secretion, and prophylactic antibiotics (cefoperazone sodium and sulbactam sodium, combined with ornidazole intravenously for 3 d). On post-procedure day 4, the temperature was 36.5°C, the C-reactive protein level was 1.9 mg/L (reference range 0.0–8.0 mg/L) and the neutrophil percentage was 63.8% (reference range 50–70%). The patient was discharged with no complications and recovered uneventfully.

### Case 2

A fifty-year-old man presented at the local hospital complaining of odynophagia after having ingested a fish bone 5 days earlier. An esophageal CT scan showed a foreign body in the esophagus at the level of the first thoracic vertebra. Gastroscopy revealed 0.5 cm of a foreign body visible above the mucosa. Conventional endoscopy was attempted at the local hospital without success and surgical dissection was recommended. The patient then visited another hospital. However, the fish bone was not found with gastroscopy. The patient had ongoing symptoms of odynophagia, and experienced pain in the left ear and face.

**Figure 2:**
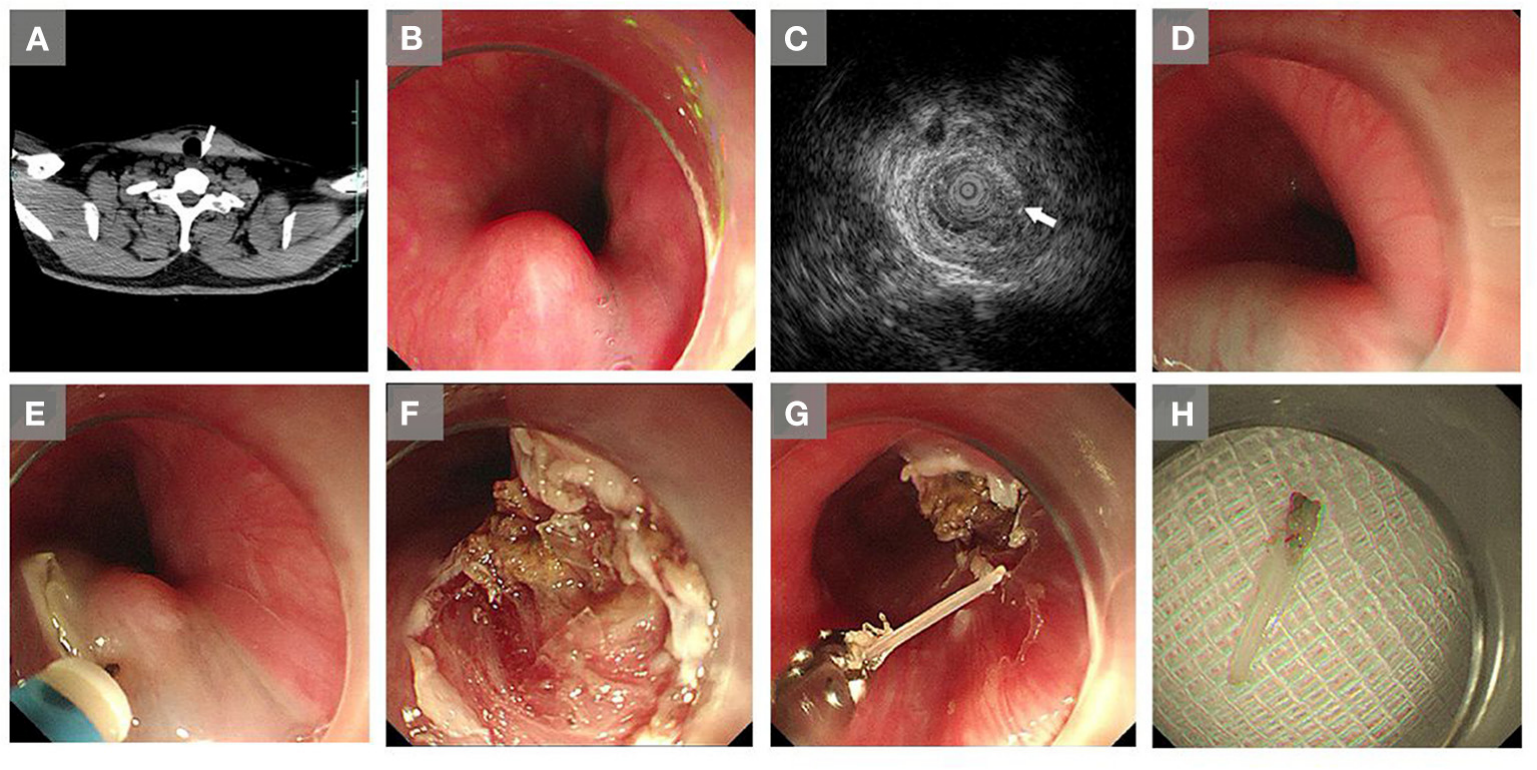
Images of the embedded foreign body in the esophagus, and extraction *via* endoscopic submucosal dissection in Case 2. **(A)** A buried foreign body (arrow) in the esophagus was revealed by a computer tomography (CT) scan at our hospital. **(B)** A mucosal protrusion was observed with endoscopy. **(C)** Intraoperative endoscopic ultrasound imaging showed a linear hyperechoic mass. **(D)** Mixture fluid was injected around the mucosal protrusion. **(E)** An incision was made under the mucosal protrusion with a dual knife. **(F)** Dissection of the mucosa and submucosa. **(G)** Removal of the foreign body (a fish bone) by forceps. **(H)** The removed fish bone.

The patient was subsequently transferred to our hospital. His medical and family history was unremarkable. The temperature was 37.0°C. Other vital signs were stable on admission. The white blood cell count was 5.61 × 10^9^/L (reference range 4.0–10.0 × 10^9^/L), and the C-reactive protein level was 0.3 mg/L (reference range 0.0–8.0 mg/L). The patient was Hepatitis B surface antigen positive. The alanine aminotransferase concentration was 42 U/L (reference range 9–50 U/L) and the aspartate aminotransferase concentration was 35 U/L (reference range 15–40 U/L). Other laboratory results were normal. An enhanced esophageal CT revealed a linear, high-density, foreign body embedded at the entry to the thorax on the left, front position of the esophagus, with a length of 0.75 cm (Figure 2A). The patient was arranged for subsequent ESD. The procedure began with endoscopic evaluation under general anesthesia with endotracheal intubation. A mucosal protrusion was identified during endoscopy (Figure 2B). Intraoperative EUS was performed and the foreign body was detected as a linear hyperechoic mass (Figure 2C). Mixture fluid was injected under the mucosal protrusion (Figure 2D), and a dual knife was applied to make the incision (Figure 2E). The mucosa and submucosa were dissected (Figure 2F). ESD steps was performed similarly as described in Case 1. Finally, the foreign body, a 1 cm long fish bone, was successfully removed with Rat Tooth forceps (Olympus) (Figures 2G,H). On post-procedure day 1, the temperature was 36.6°C. All other vital signs were stable and lab results were normal. The patient complained of mild sore throat (one out of 10 on the pain scale). The patient received proton pump inhibitors (omeprazole) for suppression of gastric acid secretion for 2 days, and was discharged without any complications and recovered uneventfully.

## Discussion

The majority of ingested foreign bodies pass through the digestive tract uneventfully; approximately 10–20% of patients need nonoperative intervention, and only a small proportion (<1%) requires surgery (5). Endoscopy-based methods have become the preferred choice for removing ingested foreign bodies in the esophagus since McKechnie et al. reported the first case of foreign body extraction by using a flexible endoscope (6, 7).

However, migration into the deeper tissue of the esophagus is a rare and serious complication of ingested foreign bodies. Removal of an embedded foreign body *via* conventional endoscopic methods can be impossible when the object cannot be directly seen with a standard endoscope (8). Surgery is required when the sharp object is impacted deeply into the esophageal wall (9). In previous studies, Cao et al. reported on a patient with an embedded fish bone in the lower esophagus which could not be extracted with the aid of a flexible endoscope, and which was subsequently removed *via* thoracoscopic surgery (8). Shahi et al. reported on a case in which a foreign body was embedded in the lower esophagus, and which was removed *via* gastric resection with resection anastomosis (7). Although modern esophageal surgery is generally safe, postoperative fistulae, strictures and long-term hospitalization may impair the quality of life for some patients, and still threaten them. The difficulty in removing embedded foreign bodies also leads to a risk-benefit medical dilemma, in which leaving the foreign object untreated can be less damaging than trying to remove it. For example, Kikuchi et al. reported on a case of a suspected fish bone embedded in the middle esophagus. The invasiveness of surgical removal was considered too great and the foreign body was left in the esophagus untreated for another year since the lesion was suspected of being benign (9).

ESD was developed as a method of endoscopic “en bloc” resection of superficial gastric cancers, and was considered as minimal invasive endoscopic therapy (3, 10, 11). The potential for performing truly scar-less, safer procedures, as well as with lower rates of complications, is appealing to both physicians and patients. Furthermore, it is easier for patients to accept removal of the foreign body *via* a natural orifice instead of *via* surgery (12). In a few recent case reports, ESD has been applied in some circumstances. For example, Li et al. presented a case in which ESD was arranged for a submucosal tumor in the stomach, which actually turned out to be a buried fish bone (13). Wang et al. reported on a case in which ESD was used to extract an esophageal foreign body embedded in the submucosal layer (14). Watanabe et al. reported on a case in which ESD was used to extract a buried metallic mesh in the mucosa of the upper esophagus of a 5-year-old boy (15).

Furthermore, early diagnosis and endoscopic intervention are paramount to reduce the potential complications of embedded foreign bodies. In the first case, the diagnosis of esophageal foreign body had not been made until the second visit to the local hospital even when the patient presented with symptoms. In some previous studies, Chung et al. reported on a case in which a two-year recurrent, deep neck infection was caused by a buried fish bone (16). Shahi et al. reported on a recent case in which a foreign body had been embedded in the lower esophagus of a patient for 6 years despite obvious signs and symptoms, due to the lack of definitive diagnostic procedures and expertise (7). When a patient complains of symptoms, such as swelling, pain in the neck, dysphagia or odynophagia, it is crucial that the physician consider the possibilities of perforation or migration of a foreign body into the esophagus, even if the object is not detected by an endoscopic examination. A high index of suspicion is necessary to make the correct diagnosis (17). A delay in obtaining the correct diagnosis can result in suboptimal outcomes.

In addition, we highly recommend esophageal CT scans to detect foreign bodies and adjacent vital blood vessels or organs. This helps the endoscopist to locate the foreign body (17). Intraoperative US guidance can detect how deeply the foreign body is embedded, and as such, is also recommended during ESD. Furthermore, we recommend submucosal injection around the mucosal protrusion, which could be the most suspicious location of a buried foreign body. A careful, mucosal incision can subsequently be made on the mucosal protrusion.

However, ESD is limited when the foreign body has completely migrated out of the esophageal wall, such as migration into the mediastinum, as the detection and removal of the foreign body become relatively difficult under endoscopic procedure. Meanwhile, bleeding, perforation and mediastinal emphysema are regarded as common perioperative complications of esophageal ESD (18, 19).

In this report, two cases were presented in which ESD was shown to be a safe and effective method in extracting a buried and covered foreign body in the esophagus. CT scans and US guidance are valuable diagnostic tools for facilitating this kind of procedure. As this study shows, foreign body migration should be taken into consideration even if a foreign body is not detected under standard endoscopy, especially when patients present with symptoms of neck pain, dysphagia or odynophagia.

## Data Availability Statement

The original contributions presented in the study are included in the article/supplementary material, further inquiries can be directed to the corresponding author.

## Ethics Statement

The studies involving human participants were reviewed and approved by the Clinical Research Ethics Committee of the First Affiliated Hospital, College of Medicine, Zhejiang University (number of approval: IIT20200447A). The patients/participants provided their written informed consent to participate in this study. Written informed consent was obtained from the individual(s) for the publication of any potentially identifiable images or data included in this article.

## Author Contributions

DL and FJ conceived of the study. DL, LL, QG, and FJ collected and analyzed the clinical history and data. DL, AJ, and BB drafted and critically revised the manuscript. All authors approved of the final version of the manuscript.

## Conflict of Interest

The authors declare that the research was conducted in the absence of any commercial or financial relationships that could be construed as a potential conflict of interest.

## Publisher's Note

All claims expressed in this article are solely those of the authors and do not necessarily represent those of their affiliated organizations, or those of the publisher, the editors and the reviewers. Any product that may be evaluated in this article, or claim that may be made by its manufacturer, is not guaranteed or endorsed by the publisher.
